# Substrate recognition and ATPase activity of the *E. coli* cysteine/cystine ABC transporter YecSC-FliY

**DOI:** 10.1074/jbc.RA119.012063

**Published:** 2020-03-06

**Authors:** Siwar Sabrialabed, Janet G. Yang, Elon Yariv, Nir Ben-Tal, Oded Lewinson

**Affiliations:** ‡ Department of Biochemistry and the Rappaport Institute for Medical Sciences, Faculty of Medicine, Technion-Israel Institute of Technology, Haifa 31096, Israel; § Department of Chemistry, University of San Francisco, San Francisco, California 94117; ¶ Department of Biochemistry and Molecular Biology, George S. Wise Faculty of Life Sciences, Tel Aviv University, Tel Aviv 6139001, Israel

**Keywords:** membrane protein, enzyme mechanism, ABC transporter, ATPase, amino acid transport, sulfur, membrane transport, cysteine/cystine import, enantiomers, YecSC-FliY

## Abstract

Sulfur is essential for biological processes such as amino acid biogenesis, iron–sulfur cluster formation, and redox homeostasis. To acquire sulfur-containing compounds from the environment, bacteria have evolved high-affinity uptake systems, predominant among which is the ABC transporter family. Theses membrane-embedded enzymes use the energy of ATP hydrolysis for transmembrane transport of a wide range of biomolecules against concentration gradients. Three distinct bacterial ABC import systems of sulfur-containing compounds have been identified, but the molecular details of their transport mechanism remain poorly characterized. Here we provide results from a biochemical analysis of the purified *Escherichia coli* YecSC-FliY cysteine/cystine import system. We found that the substrate-binding protein FliY binds l-cystine, l-cysteine, and d-cysteine with micromolar affinities. However, binding of the l- and d-enantiomers induced different conformational changes of FliY, where the l- enantiomer–substrate-binding protein complex interacted more efficiently with the YecSC transporter. YecSC had low basal ATPase activity that was moderately stimulated by apo FliY, more strongly by d-cysteine–bound FliY, and maximally by l-cysteine– or l-cystine–bound FliY. However, at high FliY concentrations, YecSC reached maximal ATPase rates independent of the presence or nature of the substrate. These results suggest that FliY exists in a conformational equilibrium between an open, unliganded form that does not bind to the YecSC transporter and closed, unliganded and closed, liganded forms that bind this transporter with variable affinities but equally stimulate its ATPase activity. These findings differ from previous observations for similar ABC transporters, highlighting the extent of mechanistic diversity in this large protein family.

## Introduction

ABC transporters comprise one of the largest protein families of any proteome and play diverse and vital roles in all kingdoms of life (1–3). These membrane-embedded enzymes use the energy of ATP hydrolysis to transport a wide range of biomolecules against their concentration gradients (4–6). In humans, genetic defects in ABC transporters lead to diseases such as Tangier disease, adrenoleukodystrophy, and cystic fibrosis (7–9), and their elevated expression underlies the phenomenon of tumor multidrug resistance (10, 11).

It is therefore of no surprise that decades of research have been dedicated to understanding ABC transporter structure and function. Despite the diversity of their physiological roles and substrate recognition profiles (12–17), ABC transporters share a common basic architecture, minimally comprising two intracellular nucleotide-binding domains (NBDs)^2^ that bind and hydrolyze ATP and two transmembrane domains (TMDs) that form a substrate translocation pathway (18–21). ATP is bound at two composite sites formed at the interface of the NBDs, and proper formation of the ATP-binding sites requires that the NBDs close into a tight head-to-tail sandwich (22). Binding and hydrolysis of ATP drives transition of the TMDs between inward- and outward-facing conformations with concomitant changes of substrate-binding affinities (23–25). Thus, for an exporter, the inward-facing conformation has the higher substrate-binding affinity, which is lowered upon transition to the outward-facing conformation, and vice versa in an importer. Binding of the substrate generally promotes closure of the NBDs and subsequent ATP hydrolysis (26–28), and this allosteric communication provides a positive feedback mechanism for substrate translocation.

ABC transporters that function as importers are found almost exclusively in prokaryotes (6, 29). The importers do not bind their substrates directly but, rather, work in concert with a substrate-binding protein (SBP). The SBP binds the substrate with high affinity, delivers it to the transporter, and largely dictates the transport specificity (20, 29, 30). Although there are exceptions (31, 32), each SBP is specific for one transporter, and together they form the functional transport unit. In bacteria, ABC importers are major determinants of high-affinity acquisition of essential nutrients (33–36). Their function becomes essential in nutrient-depleted environments; therefore, many bacterial ABC import systems are directly linked to bacterial virulence and pathogenesis (37–39).

Sulfur is an essential element for all life forms, and bacteria are no exception. It is used for synthesis of amino acids, in iron–sulfur clusters, as a redox reactant, and in coordination of transition metals such as zinc and copper (40, 41). Because of the unique chemical properties of sulfur, it cannot be readily substituted by other elements; therefore, to satisfy their sulfur quota, bacteria evolved elaborate mechanisms for sensing, acquiring, and assimilating sulfur atoms (42–45). Sulfur-containing organic compounds, such as cysteine and its oxidized dimeric form cystine, GSH, and aliphatic sulfonates, provide important sulfur sources for bacteria (42, 46). Under conditions of sulfur limitation, CysB, a LysR-type transcriptional regulator, up-regulates the expression of various uptake systems that are specific for importing sulfur-containing organic compounds (47). Among these are the ABC transport systems *tauABC*, *ssuABC*, and yecSC-*fliY*, which import taurine, aliphatic sulfonates, and cysteine/cystine, respectively (48–50). The importance of the three systems in acquiring sulfur under cysteine/sulfur starvation conditions and in redox homeostasis have been demonstrated by determining the growth phenotype of deletion strains and by uptake of a radiotracer by whole cells (49). However, our understanding of their molecular-level biochemistry remains limited, likely because of the technical challenges often associated with working with membrane proteins.

Here we describe overexpression and purification of the components of the *yecSC-fliY* ABC cysteine/cystine importer (50). Using purified components, we investigated the substrate recognition profile of FliY (the SBP) with an emphasis on discrimination between the l- and d-enantiomers of cysteine and cystine. We characterized the ATPase activity of the transporter and its modulation by the SBP and the l- and d-enantiomers. We describe a mechanism of tight coupling between ATP hydrolysis and the presence of the SBP and selective stimulation of ATP hydrolysis by the l-enantiomers.

## Results

### Recognition profile of FliY, the SBP of the system

In ABC importers, transport specificity is almost exclusively determined by the binding specificity of the SBP. The SBP binds the substrate with high affinity and delivers it to the membrane-embedded transporter (4, 5). In Gram-positive bacteria, the SBP is tethered to the membrane via a lipid anchor or fused directly to the transporter. In Gram-negative bacteria, the SBP is a soluble periplasmic protein (15, 51, 52). To study the recognition spectrum of the YecSC-FliY import system, we first overexpressed and isolated the FliY SBP. Following induction with isopropyl 1-thio-β-d-galactopyranoside (IPTG), whole-cell lysates showed dramatic enrichment of two protein bands (Fig. S1*A*). The higher band is presumably the immature form of the SBP, which includes an intact N-terminal signaling sequence. The lower band is most likely the mature SBP, in which the signal sequence is cleaved upon secretion to the periplasm. The presence of both species in whole-cell lysates suggests that the high levels of overexpression lead to overflow of the protein export machinery and accumulation of cytosolic immature FliY. Indeed, the higher molecular band was absent from the periplasmic extract, and the mature protein was subsequently purified to homogeneity by Ni-NTA chromatography (Fig. S1*B*). The purified protein was highly monodisperse in size exclusion chromatography, indicting a single molecular species that approximately corresponds in size to the monomeric form of FliY (Fig. S1*C*).

We then used two independent methods to measure substrate binding by FliY: nano differential scanning fluorimetry (nanoDSF) and isothermal titration calorimetry (ITC). nanoDSF is based on the observation that the thermal stability of a protein is increased upon ligand binding (53, 54). By exciting the protein at 280 nm and measuring the ratio of 350-nm and 330-nm fluorescence intensities while heating at a constant rate, one can determine the protein denaturation midpoint (Tm). This experiment is conducted in the absence and presence of a potential ligand, and a binding event is detected by a shift of the Tm to a higher temperature. When two different ligands induce substantially distinct bound conformations, the magnitude of the shift of the Tm differs. Thus, nanoDSF can resolve different ligand-bound conformations under saturating conditions. In contrast to nanoDSF, ITC directly measures ligand binding by measuring the amount of heat released or absorbed during a binding event. ITC is considered a benchmark method for measuring protein–ligand interactions (55, 56). Combination of these two approaches (nanoDSF and ITC) provides complimentary information regarding a protein–ligand interaction event.

Previous *in vivo* growth studies have suggested that the FliY-YecSC ABC transport system satisfies the sulfur requirements of *Escherichia coli* by importing a variety of compounds, such as the amino acid cysteine, its oxidized dimeric form cystine, djenkolic acid, and lanthionine (49, 50). We therefore studied the binding of various sulfur-containing compounds by FliY.

**Figure 1.**
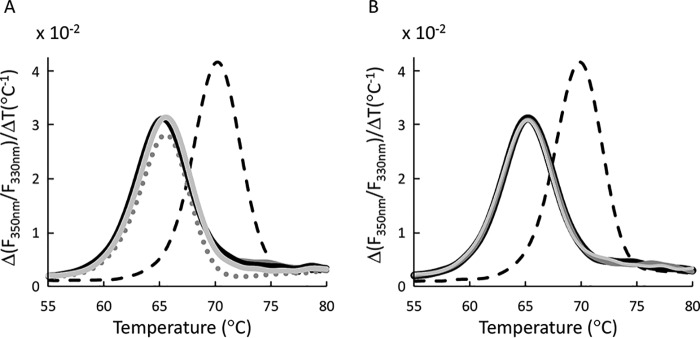
**Binding of sulfur-containing compounds by FliY.** *A*, nanoDSF measurements conducted with 30 μm FliY in the presence of 200 μm of the following: no addition (apo FliY, *solid black trace*), l-cysteine (*dashed black trace*), l-serine (*light gray*, *solid*), l-methionine (*light gray*, *dotted*), and GSH (*dark gray*, *solid*). *B*, same as in *A*; shown are the measurements for apo FliY (no addition, *solid black trace*), l-cystine (*dashed black trace*), d-cystine (*light gray, solid*), and d-cysteine (*dark gray*, *solid*). Shown are representative experiments conducted at least three times.

In the absence of ligand, FliY was a relatively stable protein with a Tm of ∼65 °C. The nanoDSF measurements were highly reproducible, as indicated by the near-perfect superimposition of replicates (Fig. S2). As expected, addition of nonrelated substrates, such as d-maltose or d-arabinose, had no thermostabilizing effect (Fig. S2). In contrast, addition of l-cysteine led to significant stabilization of the SBP by ∼4.5 °C (Fig. 1*A*). Next we tested the amino acid serine, which is identical to cysteine except for the absence of the sulfur atom from its side chain. Despite this similarity, l-serine had no thermostabilizing effect on FliY, suggesting that the sulfur atom is an important determinant of FliY recognition (Fig. 1*A*). However, other sulfur-containing compounds, such as l-methionine, GSH, and djenkolic acid, had no thermostabilizing effect, demonstrating the specificity of the FliY–l-cysteine interaction (Fig. 1*A*). Similar to l-cysteine, addition of the l-enantiomer of its oxidized dimeric form (Cys-S-S-Cys, cystine) also led to thermostabilization of FliY (Fig. 1*B*). However, for cysteine and cystine, the effect was highly stereospecific, as no thermostabilization effect was observed in the presence of d-cysteine or d-cystine (Fig. 1*B*). Taken together, the nanoDSF results suggest that FliY specifically binds the l-enantiomers of the amino acid cysteine and its oxidized dimeric form (l-cystine).

**Figure 2.**
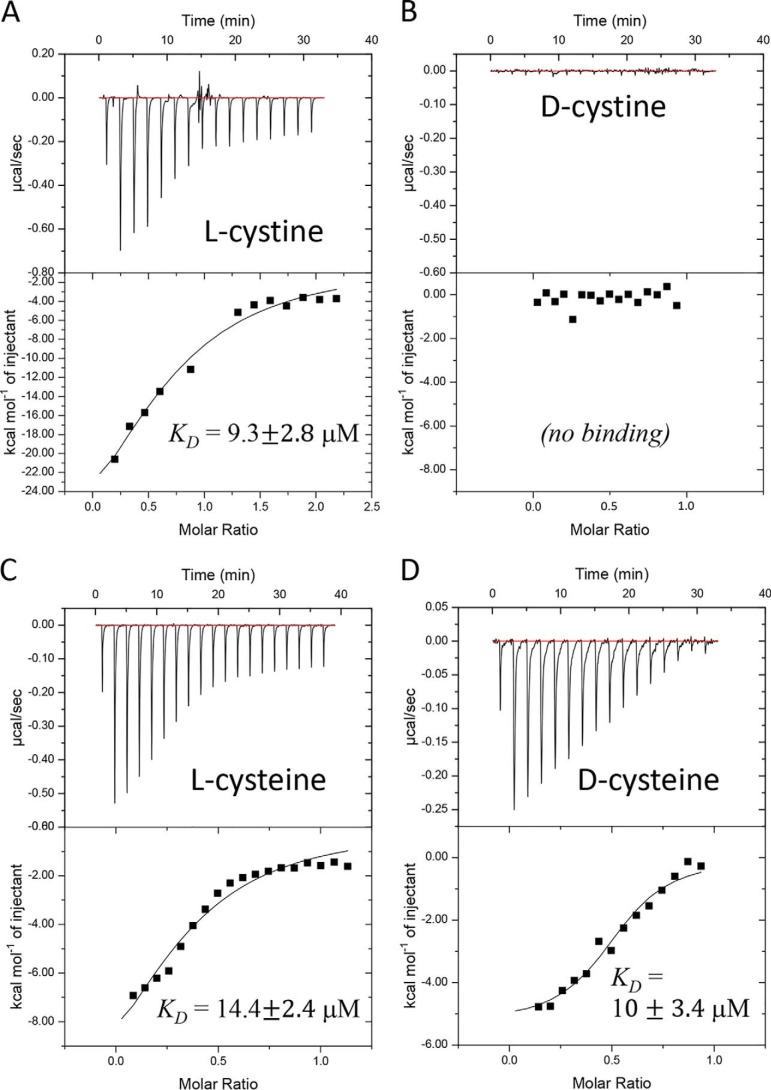
**Affinity of binding of the d/l-enantiomers of cystine and cysteine.** *A–D*, isothermal titration calorimetry was used to determine the binding of l-cystine (*A*), d-cystine (*B*), l-cysteine (*C*), or d-cysteine (*D*). Shown are consecutive injections of 2-μl aliquots from 200–400 μm solutions of the indicated amino acid into 200 μl of 70 μm FliY. The *top panels* show the calorimetric titration, and the *bottom panels* display the integrated injection heat derived from the titrations, for which the best-fit curve (*solid black trace*) was used to calculate the *K_D_*. The experiments were conducted three times, and the *K_D_* value is mean ± S.D. of three independent experiments.

Next we used ITC to measure the binding affinity of FliY to different ligands. Titration of l-cystine to apo FliY generated a strong exothermic signal (Fig. 2*A*), and a fit with a simple 1:1 interaction model yielded a *K_D_* value of 9.3 ± 2.8 μm. This binding affinity is similar to published values for other amino acid SBPs, such as the l-glutamine SBP of *Listeria monocytogenes* (*K_D_* = 4.7 μm), but considerably weaker than that reported for the *E. coli* SBPs for l-histidine (HisJ, *K_D_* = 60 nm) and l-methionine (MetQ, *K_D_* = 0.2 nm) (57–59). This variability in binding affinities between SBPs of amino acids may reflect the environmental availability of the amino acids. Binding of l-cystine by FliY was entirely enthalpy-driven, and a positive entropic value was noted in all experiments. Although we did not attempt to pinpoint the values of ΔH and ΔS, these observations are in line with the suggestion that the mobility of class II substrate-binding proteins, such as FliY, is restricted upon ligand binding (therefore leading to a decrease in ΔS). Consistent with the nanoDSF results, titration of d-cystine to apo FliY did not produce any measurable ITC signal (Fig. 2*B*). From these results, we conclude that FliY binds l-cystine but not its d-enantiomer.

Next we conducted similar experiments with the l- and d-enantiomers of cysteine. As expected, binding of l-cysteine by FliY was readily detectable by ITC (Fig. 2*C*) and was also exothermic and mainly driven by enthalpy. The affinity of FliY to l-cysteine (*K_D_* = 14.4 ± 2.4 μm) was modestly weaker (1.5-fold) than for l-cystine, but this difference was determined to be significant using a Student's two-sided *t* test (*p* = 0.02). Surprisingly, binding of d-cysteine to apo FLiY was readily detected by ITC experiments (Fig. 2*D*). The affinity of FliY to d-cysteine (*K_D_* = 10 ± 3.4 μm) was similar to the affinities measured for l-cystine and l-cysteine.

**Figure 3.**
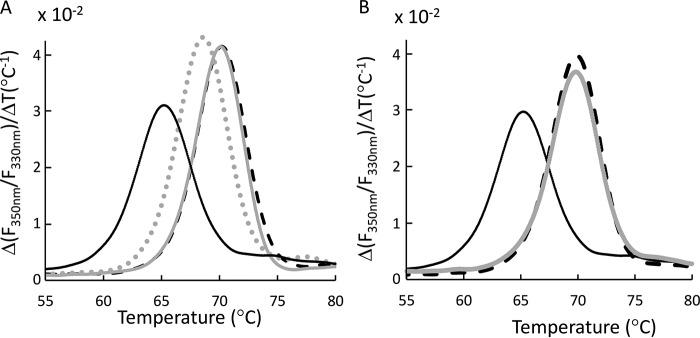
**Competition between the d- and l-enantiomers of cystine and cysteine in binding to FliY.** *A*, nanoDSF competition measurements conducted with 10 μm FliY under the following conditions: no addition (apo FliY, *solid black trace*), 50 μm l-cysteine (*dashed black trace*), 50 μm l-cysteine and 200 μm d-cysteine (*light gray*, *dotted*), and 50 μm l-cysteine and 200 μm l-methionine (*light gray*, *solid*). *B*, no addition (apo FliY, *solid black trace*), 50 μm l-cystine (*dashed black trace*), and 50 μm l-cystine and 200 μm d-cystine (*light gray*, *solid*).

With respect to binding affinity of d-cysteine, the contradiction of the ITC and nanoDSF results was puzzling. We hypothesized that d-cysteine binds at the same site as l-cysteine or l-cystine but that binding of d-cysteine induces a distinct conformational change that does not lead to increased thermostability. Recent studies have indeed demonstrated that binding of closely related substrates by SBPs can lead to different bound conformations (17, 60, 61). To explore this possibility, we conducted binding competition experiments using nanoDSF. In these experiments, a 4-fold molar excess of d-cysteine was added together with l-cysteine. We predicted that if d-cysteine binds to the same site as l-cysteine but does not stabilize FliY, then its presence will inhibit the stabilization effect mediated by binding of l-cysteine. Consistent with this prediction, relative to the presence of only l-cysteine, concomitant addition of both enantiomers led to a reproducible, ∼2 °C reduction in thermostability (Fig. 3*A*).

As a negative control, we repeated this experiment using l-methionine as a competitive ligand and did not observe a reduction in thermostability. Furthermore, competition experiments using d-cystine had no effect on the thermostabilization of FliY by l-cystine (Fig. 3*B*). Perhaps unexpectedly, the mixture of d-cysteine and l-cysteine did not lead to formation of multiple or broader peaks but, rather, to formation of a single peak of comparable width but reduced thermostability. Given the capacity of FliY to bind cystine, a putative explanation for this phenomenon may be the concurrent binding of d-cysteine and l-cysteine, which leads to an intermediate level of stabilization. Taken together, the ITC, nanoDSF, and nanoDSF-competition results suggest that FliY specifically binds l-cystine, l-cysteine, and d-cysteine and that binding of the l-enantiomers leads to a conformational change that is distinct from that induced by binding of the d-enantiomer.

### ATP hydrolysis by YecSC

ABC transporters that function as importers are divided into two classes or “types.” Type I importer systems import sugars, amino acids, and peptides (36, 62–65), whereas type II systems import metals or organo–metal complexes, such as heme, siderophores, and vitamin B_12_ (15, 66–68). The type I and type II subgroups differ structurally and mechanistically, and one distinctive mechanistic feature is their ATP hydrolysis activity. Type I ABC importers generally have low basal rates of ATP hydrolysis that are greatly stimulated by docking of the substrate-loaded SBP (69–71). In contrast, type II importers have very high basal rates of ATP hydrolysis that are much less responsive to the SBP and/or substrate (23, 34, 35, 72). To characterize the basal ATP hydrolytic activity of YecSC and its modulation by FliY, the transporter was overexpressed in *E. coli*. Following the strategy originally developed by Locher *et al.* (73), we screened multiple constructs of YecSC to identify the positions that can accommodate the His tag without interfering with membrane-embedded expression of the transporter. In this screen, we observed that tagging of the NBD at its C-terminal completely abolished its expression and that the TMD domain tolerates tagging at both termini (Fig. S3). When we compared the expression of the singly tagged constructs, the N-terminally tagged NBD showed greater expression than tagged TMD constructs (Fig. S3). Therefore, for subsequent studies, we focused on a construct where only YecC (NBD) was His-tagged, whereas YecS (TMD) was tag-free.

To extract YecSC from *E. coli* membranes, several detergents were screened. Of these, the most efficient extraction was achieved using 7-cyclohexyl-1-heptyl-β-d-maltoside, and YecSC could be subsequently purified to high homogeneity in this detergent. However, despite the clear presence of the ATPase and transmembrane domains, we could not detect any ATPase activity of 7-cyclohexyl-1-heptyl-β-d-maltoside–purified YecSC. Other detergents did not efficiently extract YecSC from membranes, and we therefore tested combinations of detergents. We found that a 1:1 (w/w) mixture of *N*-decyl-β-d-maltopyranoside (DM) and dodecyl maltoside (DDM) improved extraction of YecSC and allowed isolation of the transporter with high purity (Fig. S4). To preserve the ATPase activity of YecSC, it was necessary to add lipids to the DDM/DM-purified protein. All subsequent activity measurements were conducted in the presence of a 20:1 molar excess of purified *E. coli* polar lipids.

**Figure 4.**
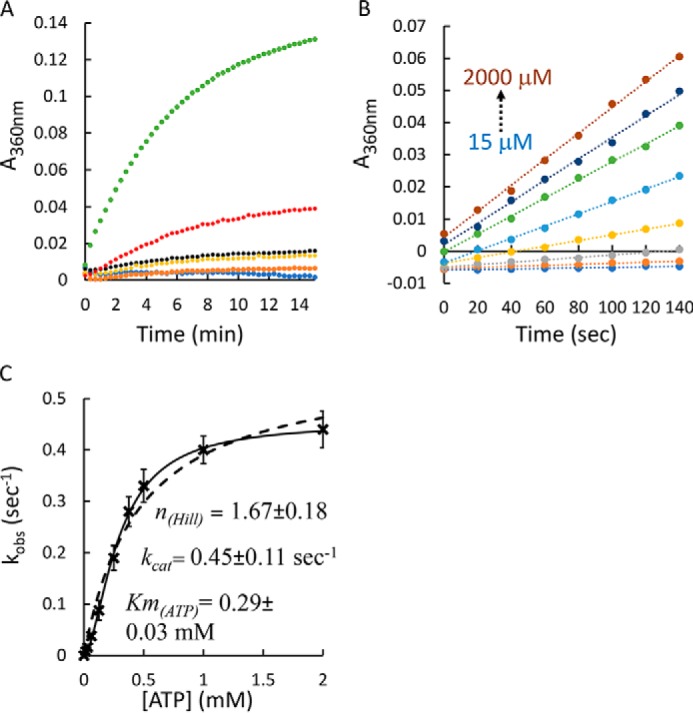
**ATP hydrolysis by YecSC.** *A*, 0.5 μm of purified YecSC was supplemented with 10 μm of *E. coli* polar lipids and incubated for 2 min with 1 mm ATP. To initiate hydrolysis, 2 mm MgSO_4_ was injected at time 0. The rate of release of P_i_ was determined by continuous monitoring of the 340-nm absorbance of the solution using the EnzCheck kit. ATP hydrolysis was measured in the presence of 0.5 μm YecSC (*black curve*); buffer only (*blue*); 1 μm FliY (*orange*); 0.5 μm YecSC and 30 μm l-cystine (*yellow*); 0.5 μm YecSC and 1 μm FliY (*red*); or 0.5 μm YecSC, 1 μm FliY, and 30 μm l-cystine (*green*). Shown are representative experiments conducted at least three times. *B*, initial rates of hydrolysis of 15–2000 μm ATP were measured in the presence of 1 μm YecSC, 5 μm FliY, and 100 μm l-cystine. *Circles* represent the experimental data, and *dotted lines* are the linear fits. *C*, the initial rates of ATP hydrolysis were plotted as a function of the ATP concentration (*crosses*). The data were then fit using the Michaelis–Menten equation (*dashed line*) or its expanded version that includes also a term for the Hill coefficient (*solid line*).

In the absence of FliY, YecSC displayed very low ATP hydrolytic activity that was barely detectable above the background level (Fig. 4*A*). Addition of l-cystine alone (in the absence of FliY) had no effect, and the ATPase activity remained near background. In contrast, addition of a 5-fold molar excess of substrate-free apo FliY led to a marked (∼3-fold) stimulation of the ATPase activity of YecSC. To rule out the possibility of contaminating ATP hydrolysis activity, we conducted experiments where FliY was present but YecSC was absent. No ATPase activity was measured in these experiments, demonstrating that the observed activity requires the presence of both YecSC and FliY. Concomitant addition of FliY and l-cystine led to the highest level of stimulation, ∼11-fold over basal activity (Fig. 4*A*).

Next, to examine the role of the two ATPase sites in YecSC, we measured the initial rates of activity under a range of ATP concentrations. As shown, at ATP concentrations of 15–2000 μm, the initial rates of ATP hydrolysis were linear for more than 2 min (Fig. 4*B*). The rate constants were plotted as a function of ATP concentration, and the data were fit using the Michaelis–Menten model or an expanded version that includes the Hill coefficient (Fig. 4*C*). Adding the term for the Hill coefficient lowered the root mean square deviation of the fit by ∼15-fold. These results suggest that the two ATP binding sites of YecSC are interdependent and hydrolyze ATP cooperatively (n_HILL_ = 1.7 ± 0.2). Similar cooperative ATP hydrolysis has been described for the vitamin B_12_ transporter BtuCD (n_HILL_ = 2), the methionine transporter MetNI (n_HILL_ = 1.7), the maltose importer MalFGK_2_ (n_HILL_ = 1.4–1.7), and the histidine importer HisPQM (n_HILL_ = 1.9) (16, 69, 72, 74). The affinity of YecSC to ATP is quite low (*K_m_*_(ATP)_ ≈ 0.3 mm), substantially weaker than that reported for BtuCD and MalFGK_2_ (10–20 μm) but similar to the *K_m_* reported for HisPQM (≈0.5 mm) and MetNI (≈0.3 mm) (16, 35, 69, 70, 72). Given the high intracellular concentrations of ATP in *E. coli*, we anticipate that YecSC would be nearly saturated with ATP under physiological conditions (75).

As shown above (Fig. 4*A*), substrate-bound FliY more strongly stimulates the ATPase activity of YecSC than substrate-free FliY. Previous work has demonstrated that class II substrate-binding proteins undergo a large Venus flytrap–like conformational change when binding substrates (76–79). This conformational change is sensed by the transmembrane domain of the transporter and provides a substrate occupancy signal that is transmitted to the nucleotide-binding domains. As a result, docking of the substrate-bound SBP stimulates ATP hydrolysis and, ultimately, transport (70). This substrate-dependent stimulation of ATPase activity can be a result of two mechanisms or their combinations. One possibility is that substrate-free and -bound FliY dock to YecSC with similar affinities, but substrate-bound FliY more efficiently induces closure of the NBDs and, thus, promotes ATP hydrolysis. Such a mechanism has been demonstrated for the ABC importers for maltose and histidine (25, 80). Alternatively, substrate binding could increase the affinity of FliY to YecSC, which leads to a higher fraction of transporter-bound FliY molecules in the ATPase assays.

**Figure 5.**
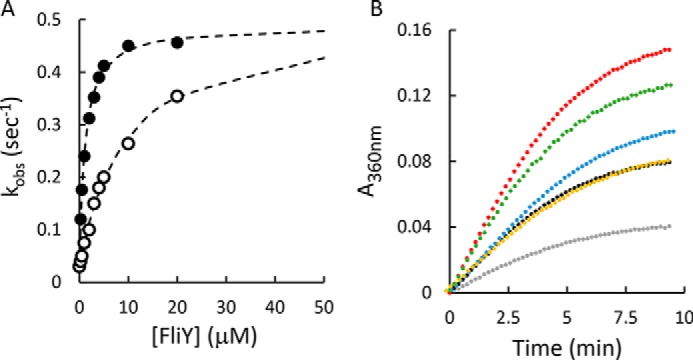
*A*, modulation of ATP hydrolysis by apo- and holo FliY. ATP hydrolysis by 1 μm YecSC was measured in the presence of a range of FliY concentrations (0.25–20 μm, as indicated) in the absence (*empty circles*) or presence (*full circles*) of 100 μm l-cystine. The *dashed line* represents the fit of the data using Michaelis–Menten. *B*, stimulation of ATPase activity by the d- and l-enantiomers. ATP hydrolysis was measured for 1 μm YecSC (*gray*); 1 μm YecSC and 2 μm FliY (*black*); 1 μm YecSC, 2 μm FliY, and 30 μm d-cystine (*yellow*); 1 μm YecSC, 2 μm FliY, and 30 μm d-cysteine (*blue*); 1 μm YecSC, 2 μm FliY, and 30 μm l-cysteine (*green*); and 1 μm YecSC, 2 μm FliY, and 30 μm l-cystine (*red*).

To discriminate between these two possibilities, we determined the initial rates of ATP hydrolysis with a range of FliY concentrations in the absence or presence of saturating l-cystine. Under both conditions, the data were readily fit with the Michaelis–Menten equation, consistent with a 1:1 FliY:YecSC interaction ratio (Fig. 5*A*). A comparison of the kinetic constants showed that the apparent *k*_cat_ was largely unaffected by the presence of substrate. In contrast, the presence of substrate lowered the apparent *K_m_* for FliY by ∼9-fold (from 10.3 to 1.1 μm). The unchanged *k*_cat_^app^ and the lower *K_m_*^app^ suggest that, when docked, apo and holo FliY equally stimulate the ATP hydrolysis activity of the transporter but that l-cysteine–bound FliY has higher affinity to YecSC than apo FliY.

In nanoDSF and ITC binding experiments with FliY, we observed that the l- and d-enantiomers of cystine and cysteine bind differently, which could lead to distinct conformations of holo FliY. In turn, this difference in conformations could influence the stimulation of ATP hydrolysis by YecSC. To test this hypothesis, we measured the stimulation of ATPase activity by each of these substrates. As anticipated based on our thermodynamic measurements, d-cystine had no effect on FliY-mediated stimulation of ATPase activity (Fig. 5*B*). This observation further supports the conclusion that FliY does not interact with d-cystine. The highest levels of ATPase stimulation were observed in the presence of the l-enantiomers of cysteine and cystine (Fig. 5*B*), suggesting a productive interaction of FliY with the l-enantiomers. Finally, FliY-d-cysteine had a modest (but reproducible) stimulatory effect that was higher than the effect of FliY alone but lower than the effect of the l-enantiomers, further supporting the hypothesis that binding of d-cysteine leads to a distinct conformational change.

### 3D structural modeling of FliY

As described above, FliY binds both enantiomers of cysteine (but not the iso-structural serine) but discriminates between the l- and d-enantiomers of cystine, binding only the former. In an attempt to understand the molecular basis of this selectivity, we employed a combination of 3D structural modeling, evolutionary analysis, and molecular docking. Notably, because cystine is twice larger than cysteine, FliY may adopt different conformations when binding each of these two ligands. We therefore used two different templates for the modeling, as described below.

Multiple sequence alignment of the query protein and its homologs facilitates homology modeling in that it may aid in finding the best structural template and in improving the query template alignment. Thus, we used HHblits (81) to search for homologs of FliY, and a search against Uniclust30 (82) yielded 250 homologs, which we aligned using MAFFT (83). We then used Modeler (84) to construct a model of FliY using the 2.26 Å resolution crystal structure of NGO2014, the l-cysteine SBP of *Neisseria gonorrhoeae* (PDB code 2YJP, 26% sequence identity to FliY (85)).

**Figure 6.**
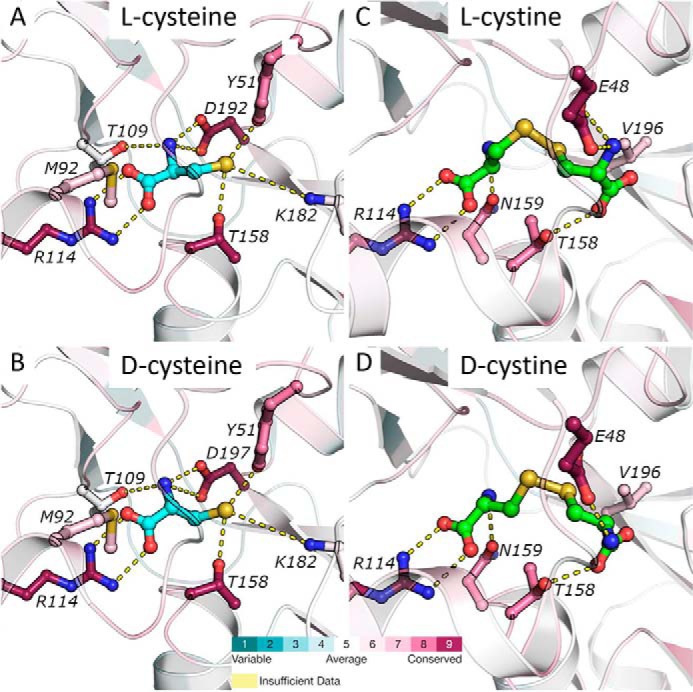
**3D modeling of FliY and enantiomer coordination.** *A–D*, FliY was modeled based on the structures of the l-cysteine SBP (*A* and *B*, PDB code 2YJP) or the l-cystine SBP (*C* and *D*, PDB code 2YLN). The protein backbone is shown as a *cartoon representation*, and selected ligand-coordinating residues are shown as *balls* and *sticks*, colored according to their ConSurf conservation score. The ligands are shown as *balls* and *sticks* and are colored *cyan* (l-cysteine, *A*; d-cysteine, *B*) and *green* (l-cystine, *C*; d-cystine, *D*). Also shown at the *bottom* is the ConSurf color-coded conservation scale (*1*, variable; *9*, conserved).

We then docked l-cysteine to this model (see “Experimental procedures” for the docking protocol) and observed that, according to the model, the C terminus of l-cysteine makes a salt bridge with the side chain of Arg-114, its N terminus makes hydrogen bonds with Thr-109 and Asp-192, and the thiol forms hydrogen bonds with Tyr-51 and Thr-158 and a weak salt bridge, 4.3Å in length, with Lys-182 (Fig. 6*A*). Docking of d-cysteine revealed that it docks in essentially the same pose as l-cysteine (Fig. 6*B*), which explains why FliY binds both enantiomers. The predicted p*K_a_* (see “Experimental procedures”) for the docked cysteine was estimated to be 6, suggesting that 95% of the bound cysteine population would be deprotonated at physiological pH. This may also explain why serine, with its much higher p*K_a_* of 15, is discriminated against; at physiological pH, serine's side chain will be protonated and will make less favorable interactions with the side chains of Tyr-51, Thr-158, and Lys-182.

We used a similar protocol and the structure of the l-cystine SBP from *N. gonorrhoeae* (PDB code 2YLN, 35% sequence identity to FliY, (85)) to predict the coordination of l-cystine by FliY. The predicted binding mode for l-cystine was very similar to what was observed in the template structure (Fig. 6*C*), whereas the pose predicted for d-cystine differed in its interaction with Glu-48 (Fig. 6*D*). The electrostatic interaction between the amine of l-cystine and the carboxylate oxygens of Glu-48 seems pivotal, as it is conserved in all FliY homologs (ConSurf grade of 9 on a scale of 1–9, (86) and also in the l-cystine SBP of *N. gonorrhoeae* (here the equivalent residue is Glu-56*).* Although the amine of l-cystine interacts with both carboxylate oxygens (Fig. 6*C*), in d-cystine, the amine is displaced and can only interact with one oxygen atom (Fig. 6*D*). This difference in binding modes may explain why FliY preferentially binds the l-enantiomer of cystine.

## Discussion

**Figure 7.**
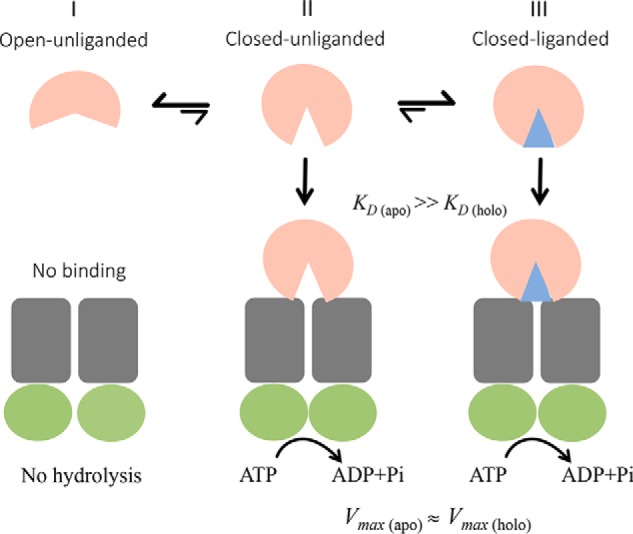
**Proposed model for the YecSC-FliY interaction and modulation of ATPase activity.** In the absence of ligand, FliY exists in a conformational equilibrium between open and closed unliganded forms, where the majority of the molecules are in the open form (*state I*). The molecules that are in state I do not interact with the transporter and do not stimulate its ATPase activity. The minority of molecules that are in the closed, unliganded form (*state II*) interact with the transporter and stimulate its ATPase activity. When ligand is present, its binding induces a population shift toward the closed, liganded form (*state III*). More molecules are not available for interaction with the transporter, and higher ATPase stimulation is observed. Nevertheless, even in the absence of substrate, when the concentrations of apo FliY are sufficiently high, the concentration of the fraction of the molecules that are in the closed, unliganded form will be higher than the *K_D_* for interaction of YecSC with the closed, unliganded FliY and also higher than the concentration of YecSC. Therefore, maximal ATPase rates are achieved (*V*_max (apo)_ ≈ *V*_max (holo)_), and further addition of substrate does not lead to increased activity. *K_D_ (apo)* and *K_D_ (holo)* represent the apparent *K_D_* for the FliY-YecSC interaction (in the absence or presence of substrate, respectively) as inferred from the apparent *K_m_* of FliY-mediated stimulation of ATPase activity.

Previous studies have suggested that SBPs of ABC transporters may exist in a conformational equilibrium between an open, unliganded form (O); a closed, unliganded form (C); and a closed, liganded form (C·L) (17, 61, 87). The results we present here for YecSC-FliY are consistent with such a model (Fig. 7).

In the absence of ligand, FliY predominantly adopts the O conformation, which does not bind to YecSC. The small fraction of molecules that are in the C conformation are available for docking to YecSC and stimulate its ATPase activity. In the presence of ligand, the conformational equilibrium is shifted toward the (C·L) conformation. More molecules are now available for docking to YecSC, and higher ATPase stimulation is observed. This is why the affinity of FliY to YecSC appears to be higher in the presence of ligand. However, it is important to note that, in terms of ATPase stimulation, the C and C ·L conformations are equivalent. At high enough concentrations, APO-FliY stimulates the ATPase activity of YecSC just as well as holo FliY (Fig. 5*A*). The only effect of substrate is to shift the equilibrium between the O and C states. This is different from what has been suggested for the maltose transporter, where the SBP and maltose are required to induce closure of the NBDs (70). In MalFGK, the ligand (maltose) has a direct role in allosteric communication via its interaction with residues in the transmembrane domain (88, 89). This substrate-mediated direct effect seems to be missing in YecSC-FliY because full stimulation of ATP hydrolysis can also be achieved in the absence of ligand. Binding of d-cysteine seems to lead to a distinct ligand bound form, C*·L, with different thermostability and a reduced ATPase-stimulatory effect. A recent single-molecule study suggested that binding of cognate and noncognate substrates by SBPs lead to productive and nonproductive conformational changes, respectively (61). This may indicate that, although d-cysteine is bound by FliY, it is not transported by YecSC or transported with reduced efficiency. This issue remains to be resolved by transport assays.

On one hand, the results we report here for the cysteine/cystine importer YecSC-FliY are very similar to those reported for the histidine ABC importer HisPQM-J (69); both systems hydrolyze ATP cooperatively with very similar Hill coefficients and nearly identical affinity to ATP. However, the effect of ligand is reversed in the two systems. In HisPQM, apo and holo HisJ bind to the transporter with equal affinities, but the *V*_max_ of ATP hydrolysis is ∼13-fold higher in the presence of histidine (69). The opposite is true for YecSC-FliY, where substrate increases the affinity of the SBP to the transporter by ∼9-fold but has no effect on the *V*_max_ of ATP hydrolysis. These differences further demonstrate the extent of mechanistic diversity in the superfamily of ABC transporters (4).

An additional difference between YecSC-FliY and other ABC transporters of amino acids is related to the complete absence of cysteine from the amino acid sequence of YecSC-FliY and other cysteine import systems. The same cannot be said for glutamine, histidine, or methionine, which are routinely found in the amino acid sequences of the ABC importers that import them. This means that, even when the intracellular level of cysteine is low, up-regulated biogenesis of YecSC-FliY can be fulfilled, leading to replenishment of the cysteine pool.

Furthermore, the YecSC-FliY system is distinct in the selectivity of the SBP. Relative to FliY, other SBPs of amino acids display much higher discrimination in favor of the l-enantiomer. For example, GlnP of *L. monocytogenes* and HisJ of *E. coli* bind only the l-enantiomers of glutamine or histidine, respectively (57, 90). Similarly, the affinity of MetQ to l-methionine is ∼15,000-fold higher than to d-methionine (91). In comparison, the affinity of FliY to l-cysteine is only ∼3-fold higher than to d-cysteine. Why would FliY be more permissive toward the d-enantiomer? FliY expression is induced under conditions of limited sulfur availability (92), and *E. coli* contains several enzymes dedicated to utilization of d-cysteine as a sulfur source, including d-cysteine desulfhydrase (93). This observation suggests that a main goal of cysteine import systems is to deliver the sulfur atom in addition to a proteogenic precursor. In this respect, d-cysteine contains the precious sulfur atom just the same and, to ensure sufficient supply of sulfur, bacteria may have evolved to also import the nonproteogenic d-enantiomer.

## Experimental procedures

### Bacterial strains and plasmids

The genes for *yecC* (ACC P37774), *yecS* (ACC P0AFT2), and *fliY* (ACC P0AEM9) were PCR-amplified from the *E. coli* K-12 derivative strain BW25113. All restriction sites for subcloning were inserted at this stage. *fliY* was inserted into the NdeI/XhoI sites of a pET21b expression vector, resulting in C-terminal fusion of a His_6_ tag. *yecS* and *yecC* were inserted in tandem into a custom-made pET-derived vector where each gene is preceded by a T7 promoter and a ribosome binding site. The YecSC construct used in this study contained an enterokinase cleavage site followed by a His_10_ tag fused to the N-terminal of YecC. *E. coli* strain DH5α (Invitrogen) was used for cloning procedures, and BL21-Gold (DE3, Stratagene) was the host for protein expression.

### Protein expression and purification

For small-scale expression testing, 20-ml cultures were grown in glycerol-supplemented Terrific Broth medium to an *A*_600_ of ∼2 and induced for 1.5 h with 0.5 mm isopropyl 1-thio-β-d-galactopyranoside. Membranes were prepared by disrupting the cells by sonication, debris removal was performed by centrifugation for 10 min at 10,000 × *g*, and membrane sedimentation was done by ultracentrifugation at 120,000 × *g* for 45 min. The His-tagged protein content of the membrane fractions was visualized using standard SDS-PAGE and immunoblot detection using an anti-His antibody. To visualize expression of FliY, cells were disrupted as above, debris was removed, and 30–50 μg of the total cell lysate was separated by SDS-PAGE and stained with Coomassie Brilliant Blue.

### Purification of FliY

Osmotic shock extracts prepared from cells overexpressing FliY in 50 mm Tris-HCl (pH 7.5), 250 mm NaCl, and 20 mm imidazole (pH 8) were loaded overnight onto a 5-ml Ni-NTA affinity column (HisTrap HP, GE Healthcare). The column was washed with 20 column volume (CV) of 20 mm imidazole before elution with a gradient of 60–250 mm imidazole. Imidazole was removed using a Sephadex G-25 column, and FliY was concentrated using Amicon Ultra concentrator (Millipore) with a molecular cutoff of 30 kDa to 5–6 mg/ml. Aliquots of FliY were snap-frozen in liquid nitrogen and stored at −80 °C until use.

### Purification of YecSC

For preparation of the membrane fraction, cells were resuspended in 50 mm Tris-HCl (pH 7.5), 0.5 m NaCl, 30 μg/ml DNase (Worthington), one complete EDTA-free protease inhibitor mixture tablet (Roche), 1 mm CaCl_2_, and 1 mm MgCl_2_ and tip-sonicated for 30 min prior to rupture by three passages in an EmulsiFlex-C3 homogenizer (Avestin). Debris was removed by 30-min centrifugation (4 °C, 10,000 × *g*). Membranes were pelleted by ultracentrifugation at 160,000 × *g* for 1 h; washed; resuspended in 50 mm Tris-HCl (pH 7.5), 0.5 m NaCl, and 10% (v/v) glycerol; and stored at −80 °C until use.

To solubilize the membranes, DM and DDM were added to a final concentration of 0.5% (w/w). The suspension was gently tilted at 4 °C for 1 h, and the insoluble fraction was removed by ultracentrifugation at 160,000 × *g* for 1 h. The soluble fraction was loaded onto a 5-ml Ni-NTA column as described above for FliY running Tris-HCl (pH 7.5), 0.5 m NaCl, 0.05% DDM, and 0.05% DM. The column was washed with 20 CV of the same buffer containing 20 mm imidazole, followed by a 10 CV wash with buffer containing 60 mm imidazole. YecSC was eluted using an imidazole gradient of 60–250 mm. Imidazole was removed by desalting, and protein was concentrated to ∼1 mg/ml using an Amicon Ultra concentrator (Millipore) with a molecular cutoff of 100 kDa. Aliquots of YecSC were snap-frozen in liquid nitrogen and stored at −80 °C until use.

### nanoDSF measurements

To remove potential copurified endogenous ligands, purified FliY was dialyzed overnight (two buffer replacements) against a 1000-fold excess of 50 mm Tris-HCl (pH 7.5) and 250 mm NaCl. The dialysis buffer was used to dilute the stock solutions of the tested ligands. FliY was incubated with different ligands, and measurements were performed with Prometheus NT.48 (Nanotemper). The tryptophan residues of the protein were excited at 280 nm, and the fluorescence intensity was recorded at 330 and 350 nm. The temperature of the measurement compartment increased from 25 °C to 95 °C at a rate of 1 °C min^−1^.

### ITC

Prior to experiments, FliY was dialyzed overnight against a 1000-fold (2 buffer replacements) volume of 50 mm Tris-HCl (pH 7.5) and 0.5 m NaCl. To avoid buffer mismatch, this dialysis buffer was used to dilute the stock solutions of the tested ligands. Calorimetric measurements were performed with the MicroCal iTC200 system (GE Healthcare), and all measurements were carried out at 25 °C. 2-μl aliquots from a 200–400 μm ligand solution (as indicated) were added by a rotating syringe to the reaction well containing 200 μl of 70 μm FliY. Data fitting was performed with Origin software using a simple 1:1 binding model, where the ligand-free form of the protein is in equilibrium with the bound species.

### ATPase assays

ATP hydrolysis was performed using the EnzChek® Phosphate Assay Kit (Molecular Probes). The reaction buffer contained 50 mm Tris-HCl (pH 7.5), 0.5 m NaCl, 0.05% DDM, 0.05% DM, 20 μm *E. coli* polar lipids, 0.2 mm 2-amino-6-mercapto-7-methylpurine riboside, 1 unit/ml purine nucleoside phosphorylase, and the indicated concentrations of ATP, YecSC, and FliY. Measurements were conducted at 37 °C in an automated plate reader (Infinite M200 Pro, Tecan). Following 2- to 5-min incubation at 37 °C, 2 mm MgCl_2_ was injected to initiate ATP hydrolysis.

### Homology modeling

Multiple sequence alignment of the query protein and its homologs facilitates homology modeling in that it may aid in finding the best structural template and in improving the query template alignment. Thus, we used HHblits (81) to search for homologs of the *E. coli* cysteine-binding protein (FliY, SWISSPROT P0AEM9). A search against Uniclust30 (82) yielded 250 homologs, which we aligned using MAFFT (83). Using the 2.26 Å resolution crystal structure of NGO2014, the cysteine binding protein of *N. gonorrhoeae* (85) (PDB code 2YJP, 26% sequence identity to FliY) and the 1.12 Å resolution crystal structure of NGO0230, the cystine binding protein from the same bacterium (PDB code 2YLN, 35% sequence identity to FliY) as templates, we constructed homology models using Modeler (84).

### Molecular docking

Prior to any docking simulations, we had to prepare the homology models and template structures for docking using the protein preparation wizard (94). We mostly used the recommended settings for the preparation, except for the minimization, which was restricted to the hydrogen atoms; the heavy atoms were maintained in their crystal structure coordinates. The ligands were prepared using LigPrep (95) (Schrödinger LLC), which generated probable protonation states at pH 7.0 ± 2.0. In this pH range, serine had a single protonation state (zwitterion with neutral side chain), whereas the cysteine had two (protonated and deprotonated side chain). Using Glide (96), we defined the receptor grid as a box with 10 Å edges, centered around the ligand coordinates from the template structure. We then used the standard precision Glide docking protocol and generated up to five docking poses per ligand.

### pK*_a_* calculations

To determine the p*K_a_* of the bound cysteine and serine ligands, we used the DelPhiPKa web server (97), which calculates an amino acid's p*K_a_* in the context of the protein environment. We used the default settings to calculate the p*K_a_* of all titratable residues, including serine, tyrosine, threonine, and cysteine. Heteroatoms were removed, excluding the cysteine ligand, which was treated as part of the protein.

### Conservation of coordinating residues

Amino acid conservation grades were calculated for the homology models and the template structures (PDB codes 2YJP and 2YLN) using the ConSurf web server (86) with default settings, except for the number of collected homologs, which was increased to 300.

## Author contributions

S. S., E. Y., and N. B.-T. data curation; S. S. and E. Y. formal analysis; S. S., N. B.-T., and O. L. investigation; S. S., N. B.-T., and O. L. methodology; S. S. and O. L. project administration; S. S., J. G. Y., and N. B.-T. writing-review and editing; J. G. Y. and O. L. writing-original draft; N. B.-T. and O. L. supervision; O. L. funding acquisition.

## Supplementary Material

Supporting Information

## Footnotes

2 The abbreviations used are:

NBD: nucleotide-binding domain
TMD: transmembrane domain
SBP: substrate-binding protein
Ni-NTA: nickel-nitrilotriacetic acid
nanoDSF: nano differential scanning fluorimetry
ITC: isothermal titration calorimetry
Tm: denaturation midpoint
DM: *N*-decyl-β-d-maltopyranoside
DDM: dodecyl maltoside
CV: column volume.
