# Supplementary material for: Substrate recognition and ATPase activity of the E. coli cysteine/cystine ABC transporter YecSC-FliY
Source: J Biol Chem. 2020 Mar 6;295(16):5245–56. doi: 10.1074/jbc.RA119.012063 (PMC7170509; doi:10.1074/jbc.RA119.012063)
Supplement: Supporting Information [file supp_295_16_5245__index.html]

Substrate recognition and ATPase activity of the E. coli cysteine/cystine ABC transporter YecSC-FliY — The E. coli cysteine/cystine ABC transporter YecSC-FliY — Substrate recognition and ATPase activity of the E. coli cysteine/cystine ABC transporter YecSC-FliY — The E. coli cysteine/cystine ABC transporter YecSC-FliY — Supporting Information 

# Substrate recognition and ATPase activity of the *E. coli* cysteine/cystine ABC transporter YecSC-FliY

## Supporting Information

- Supporting Information (to be published online) - Supporting Information figures and legends
