# Supplementary material for: Substrate recognition and ATPase activity of the E. coli cysteine/cystine ABC transporter YecSC-FliY
Source: J Biol Chem. 2020 Mar 6;295(16):5245–56. doi: 10.1074/jbc.RA119.012063 (PMC7170509; doi:10.1074/jbc.RA119.012063)
Supplement: Supporting Information [file supp_RA119.012063_157363_2_supp_485717_q6m0ys.pdf]

Substrate recognition and ATPase activity of the E. coli cysteine/cystine ABC transporter YecSC-FliY

Siwar Sabrialabed [1], Janet G. Yang[2], Elon Yariv[3], Nir Ben-Tal[3], and Oded Lewinson[1].

[1] Department of Biochemistry and the Rappaport Institute for Medical Sciences, Faculty of Medicine, The Technion-Israel Institute of Technology, Haifa, Israel
[2] Department of Chemistry, University of San Francisco, San Francisco, CA
[3] Department of Biochemistry and Molecular Biology, George S. Wise Faculty of Life Sciences, Tel Aviv University, Tel Aviv, Israel

For correspondence: lewinson@technion.ac.il

Supporting Information Figures 1-4 are included

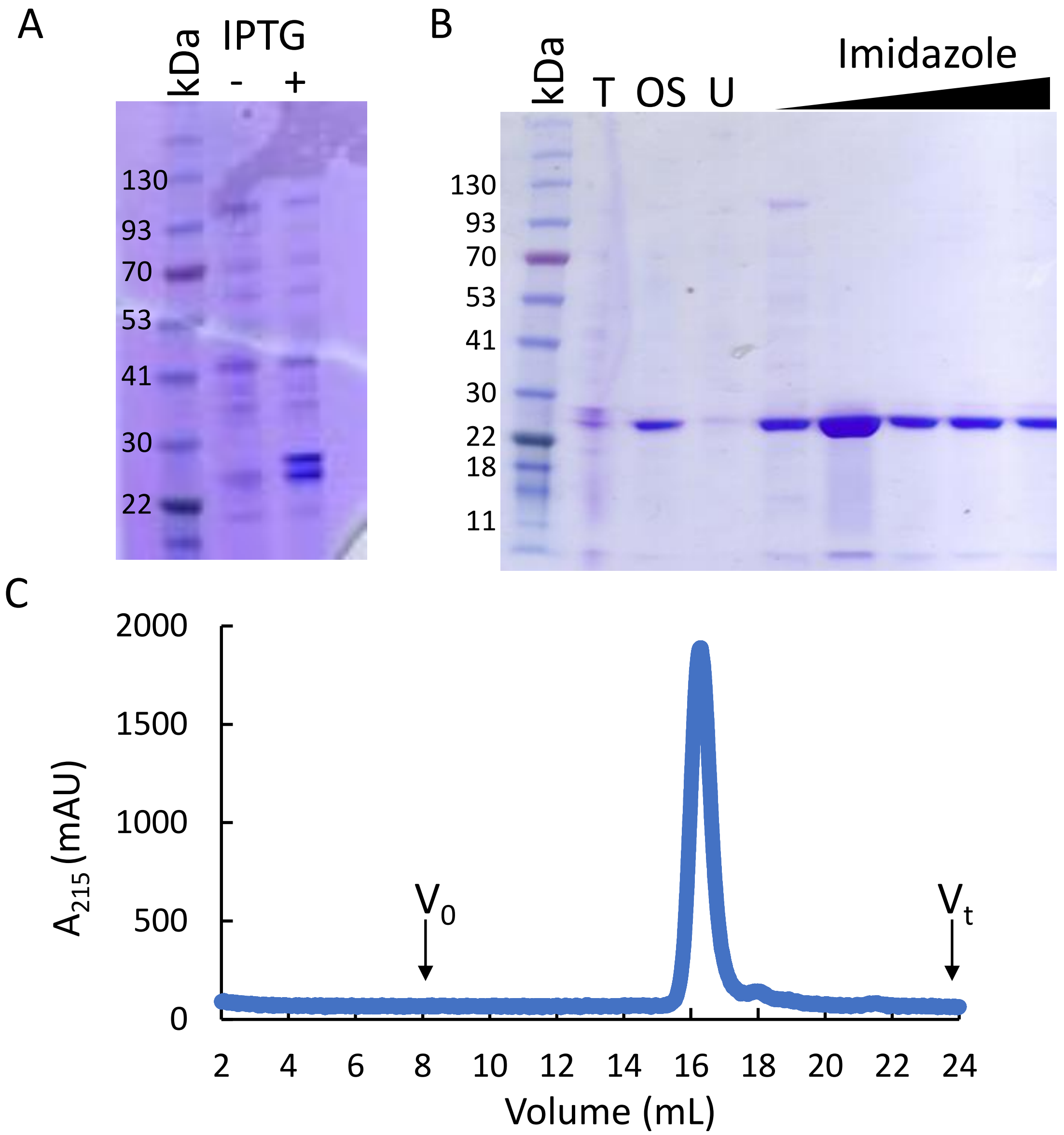

SI Figure 1. Overexpression and purification of FliY. (A) Coomassie staining of SDS-PAGE of whole-cell lysates prepared from cells transformed with a plasmid encoding FliY-His before or after the addition of 1 mM IPTG, as indicated. The two prominent proteins that appear following induction with IPTG (MW 22-30 kDa) correspond to the expected sized of mature and immature FliY. (B) Metal affinity purification of FliY. Shown is the Coomassie staining of SDS-PAGE of whole-cell lysates (T), osmotic shock extract (OS), column unbound material (U), and fractions eluted with 60-200 mM imidazole. (C) Purified FliY fractions were pooled, concentrated to ~6 mg/mL, and 50 mg Fliy were subjected to size exclusion chromatography using a Superdex200 increase 24 mL gel filtration column. V_0 and V_t indicate the column void and total volumes, respectively.

SI Figure 1

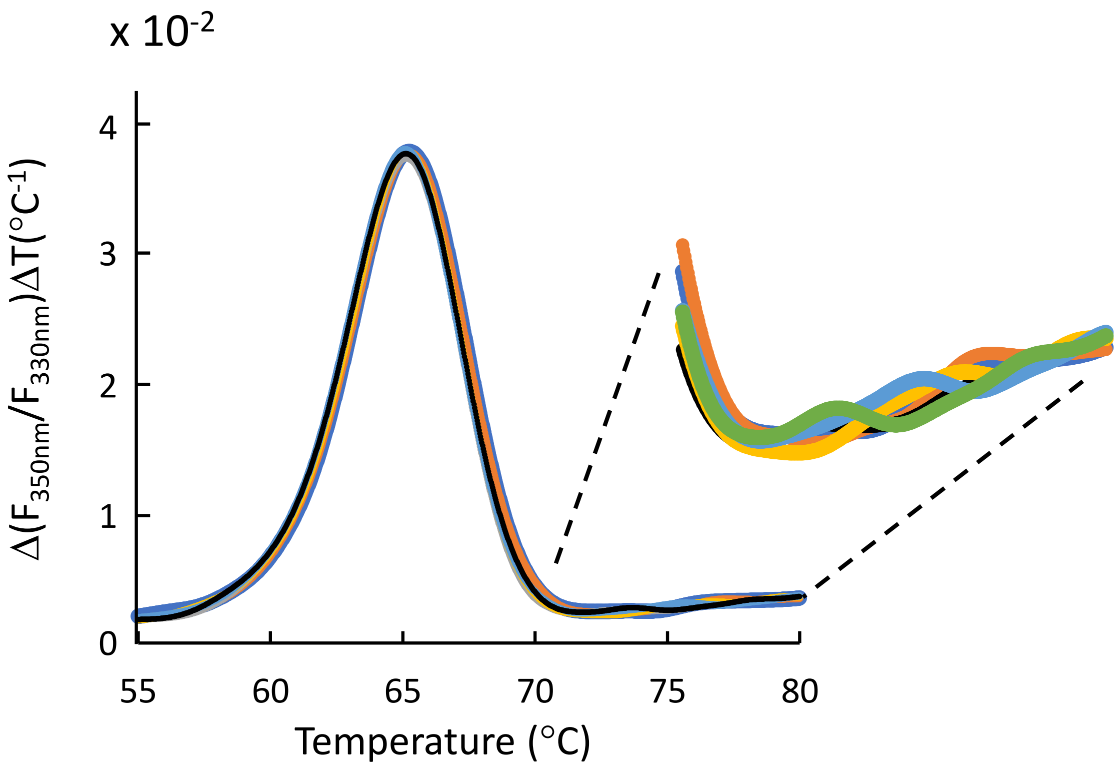

SI Figure 2. Reproducibility of the nanoDSF measurements. Shown are six individual measurements. Two measurements of apo 30 mM FliY (black, grey), two measurements of 30 mM mM FliY plus 200 mM L-arabinose (blue, green), and two measurements of 30 mM mM FliY plus 200 mM L-maltose (yellow, orange). The zoomed section highlights the presence of the multiple measurements that perfectly superimpose in the peak area.

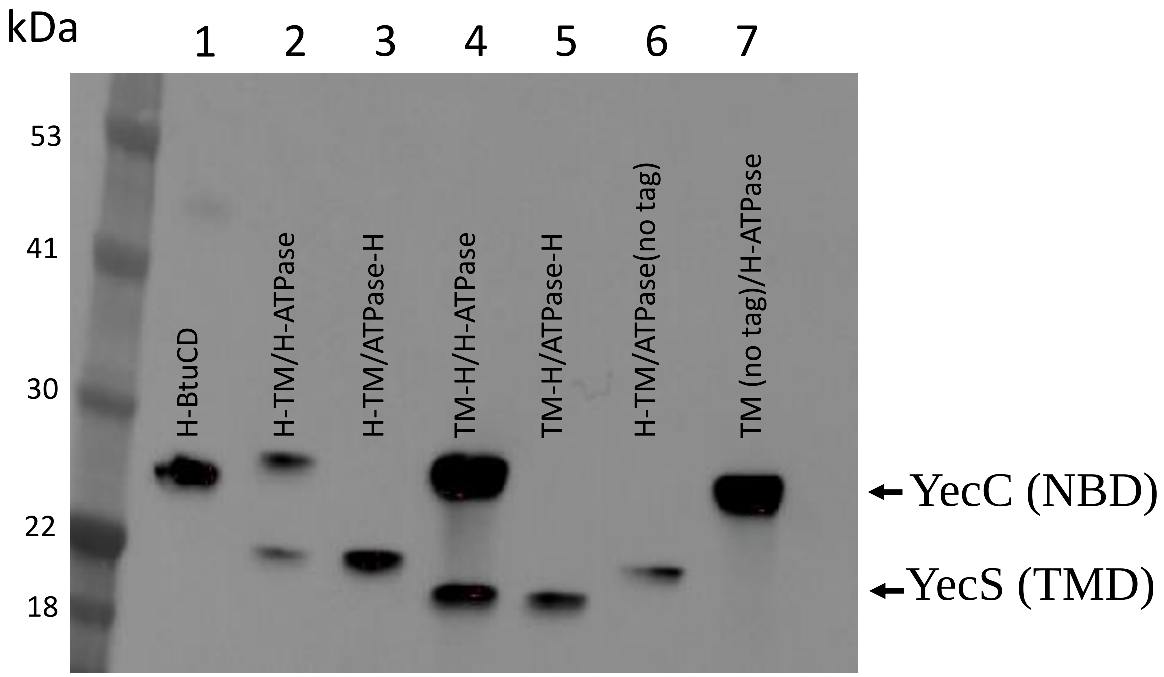

SI Figure 3. Expression screening of YecSC constructs. Shown is the immunoblot of SDS-PAGE of 1 mg of membrane fractions prepared from cells expressing different variants of His-tagged YecSC. BtuCD (lane 1) is used as a benchmark for desirable expression level. Initially, the four possible combinations of tag locations are tested (N-terminus vs. C-terminus on both proteins lanes 2-5). Note that the N-terminus tag is slightly bigger since it additionally contains a protease cleavage site. For this reason, the N-terminally tagged variants migrate as a larger molecular weight species (compare for example the N- and C-terminally tagged variants of YecS, lanes 3 and 4 respectively). Next, singly-tagged variants are prepared according the results of the initial set of dually-tagged variants. In the case of YecSC the singly tagged variants are N-terminally His-tagged YecS and no tag on YecC (lane 6), and untagged YecS and N-terminally His-tagged YecC (lane 7).

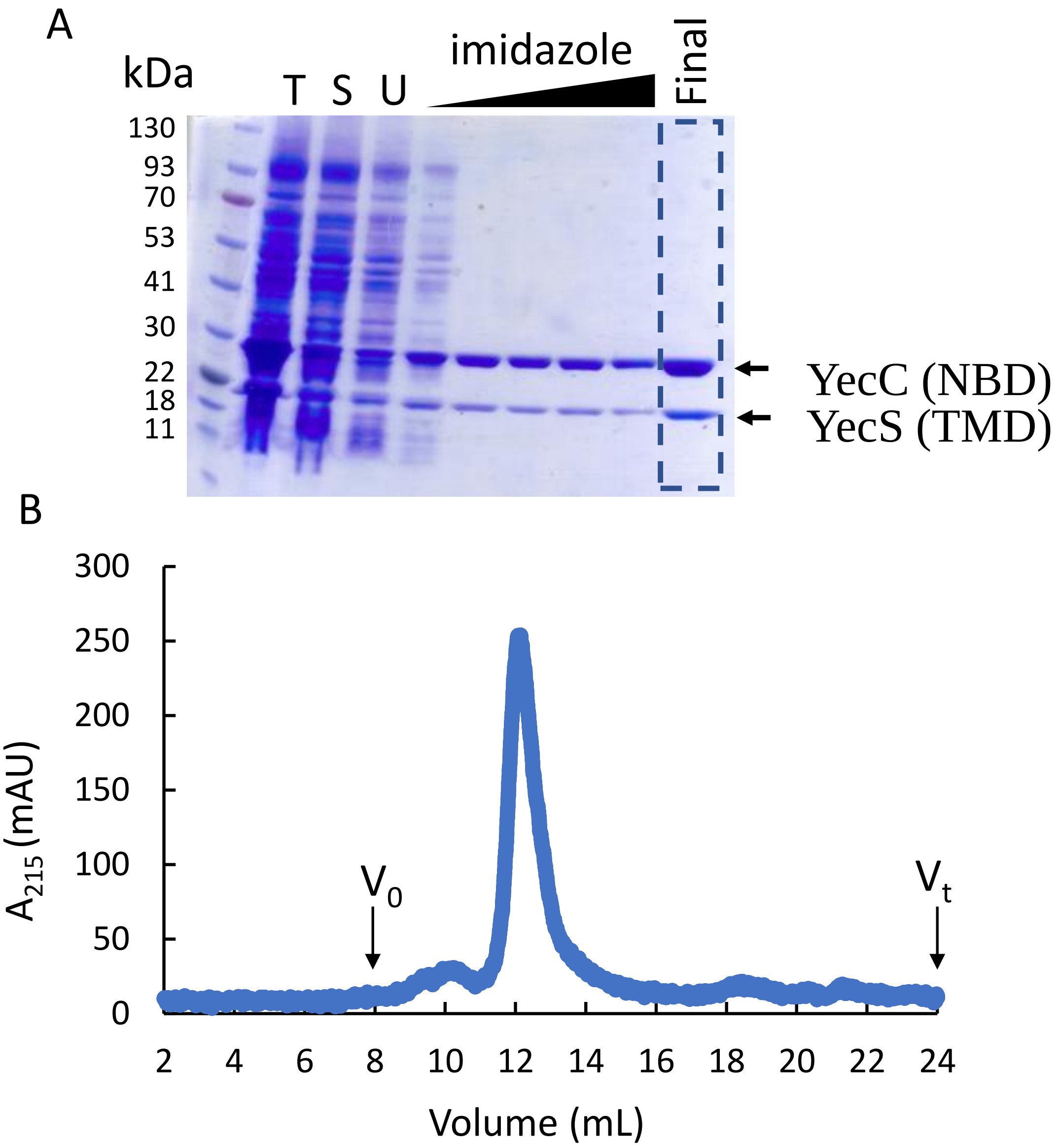

SI Figure 4. Purification and mono-dispersion of YecSC. (A) Coomassie staining of SDS-PAGE the steps of Ni-NTA purification of the complex comprised of untagged YecS and N-terminally His-tagged YecC. Shown are samples of the total membrane-fraction preparation (T), soluble fraction following membrane extraction with a mixture of DDM and DM (S), column unbound material (U), and fractions eluted with 60-250 mM imidazole. Purified fractions were pooled, concentrated to ~1 mg/mL, and the final product is highlighted by dashed box (B) The imidazole was removed using a Sephadex G-25 desalting column and 50 mg purified and desalted YecSC were subjected to size exclusion chromatography using a Superdex200 increase 24 mL gel filtration column. V_0 and V_t indicate the column void and total volumes, respectively.

SI Figure 4
